# Emergy Analysis of Biogas Systems Based on Different Raw Materials

**DOI:** 10.1155/2013/415812

**Published:** 2013-02-14

**Authors:** Yang Wang, Cong Lin, Jing Li, Na Duan, Xue Li, Yanyan Fu

**Affiliations:** ^1^College of Water Resources and Civil Engineering, China Agricultural University, Beijing 100083, China; ^2^China Zhongyuan Engineering Corporation, Beijing 100191, China

## Abstract

Environmental pollution and energy crisis restrict the development of China, and the utilization of renewable technology is an effective strategy to alleviate the damage. Biogas engineering has rapidly developed attributes to solve environmental problems and create a renewable energy product biogas. In this paper, two different biogas plants' materials were analyzed by emergy method. One of them is a biogas project whose degraded material is feces (BPF system), and the other is the one whose degraded material is corn straw (BPC system). As a result, the ecological-economic values of BPF and BPC are $28,300/yr and $8,100/yr, respectively. Considering currency, environment, and human inputs, both of the biogas projects have the ability of disposing waste and potential for development. The proportion of biogas output is much more than fertilizer output; so, fertilizer utilization should be emphasized in the future. In comparison, BPF is better than BPC in the aspects of ecological-economic benefits, environmental benefits, and sustainability. The reason is the difficulty of corn straw seasonal collection and degradation. Thus it is proposed that BPC should be combined with the other raw materials.

## 1. Introduction

China is the world's largest developing country with a population over than 1.3 billion. With the development of economy and industrialization, the problems of excessive energy consumption significantly affect the future of China. Finding new energy sources to replace fossil fuels is an urgent task for China [1–5]. Anaerobic digestion is one of the most appropriate technologies to solve these problems. There are large biomass resources in China, especially crop straw, forest residue, livestock and poultry manure, and various kinds of municipal and industrial organic wastes and waste water [2]. Such sufficient materials promote the development of biogas, especially the large-and medium-sized biogas projects.

**Figure 1:**
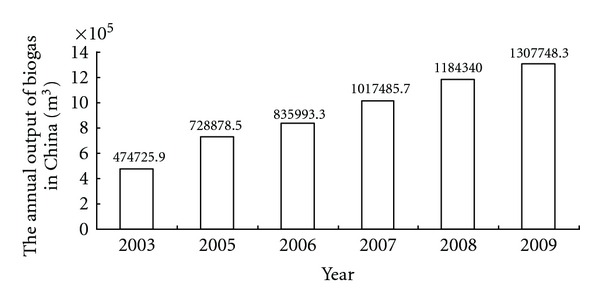
Bar chart of the number of biogas projects in China.

In 2003, the “agricultural ecological” program was carried out, which created a favorable condition for the development of biogas projects. According to statistics (shown in Figure 1), biogas yield in 2009 is 1.75 times more than 2003. In addition, the development of biogas projects also positively influences related industries, such as processing manufacturing, construction materials, and construction engineering. All of them have achieved good economic, social, and other comprehensive benefits [6].

With the number of biogas projects increasing, biogas ecological systems have attracted more and more attention. Biogas ecological system is a complexity composed of agricultural, environment, energy, society, and other relevant sectors. The aims of a biogas ecological system are producing an energy carrier from renewable resources and achieving multiple environmental benefits. Some research has been taken on biogas ecological systems, but mostly, the focus have been on the technical evaluation, economic benefit, and energy flows [7–13]. The research methods of assessing biogas ecological system include exergy accounting, energy analysis, life cycle assessment, and emergy analysis which have been developed in the last 30 years. Emergy is chosen in this paper because it is a particularly appropriate tool to evaluate the agricultural production system. Emergy focuses on the compound ecosystem at the interface between natural and human systems, which involves many subject areas, not only the systems of ecology, ecosystem ecology, energetic, resource science, environmental science, systematic, earth science, and other natural sciences, but also those relating to economics, sociology, forecasting and the other humanities [14, 15]. Emergy method unifies all the measures, and the whole inputs indices along the forming process are converted into solar emergy (sej) and solar transformation (sej/J). So, both the environmental values and the economic values can be calculated, which simplifies the assessment process.

All the research mentioned earlier is focused on one kind of biogas plant, and the material used as input is not differentiated. However, with the development of biogas technology, there are several biogas materials species. At present, the main anaerobic fermentation materials are straw and feces, and their fermentation process and parameters (temperature, concentration, refluxing ratio, stir mode, etc.) are very different. Therefore, only by considering the different characters of materials, biogas project system analysis could be comprehensive and correct. In this paper, two representative biogas projects are chosen to be compared, and emergy is used to analyze their characteristics. The aim of this study is to assess the ecological-economic benefit, environmental benefit, and sustainable development capability of two different raw materials (corn straw and feces) in the biogas generation system and lay a theoretical foundation for the generalization of the ecological biogas production system. All the calculations aim to be as transparent as possible in order to make the results useful for future analyses.

## 2. Materials and Methods

### 2.1. Study Site

The two biogas projects studied in this paper are different in degraded materials; one is corn straw, and the other is feces. All the data come from the results of site investigation among the whole year. 

#### 2.1.1. BPF System

The biogas project, where degraded material is feces (BPF), is located in the west of Hegezhuang village of Chaoyang district in Beijing. The total investment of biogas plant is RMB 2,600,000 Yuan, occupying 2930 m^2^. BPF began operating in October of 2010, processing feces from three villages, Naidong, Hegezhuang, and Naixi. This biogas project solved the problem of feces pollution and improved the quality of village life.

The technology of BPF is continuous stirred tank reactor (CSTR), and the material of the tank is enameled pressed steel. The capacity of the BPF is 400 m³, which has a liquid-gas storage integrated tank, including 224 m³ liquid storage volume and 200 m³ gas storage volumes. The biogas project can digest 10~20 t solid manure and 25 t domestic sewage per day, and its biogas yield is 650 m³/d. In routine management, the fermented concentration is maintained to 6%~10% by the way of biogas slurry refluxing. A solar system is one of the most important parts of the whole biogas plant, because it can increase feeding materials temperature to 5°C in winter. Moreover, some of the output biogas is heated to maintain fermented temperature. In spring and autumn, 250 m³ biogas will be heated, and the output is 400 m³. In summer, because of high temperature, it is not necessary to heat the biogas tank; so, the entire biogas of 650 m³ will be exported. In winter, there is 350 m³ biogas heated and 300 m³ biogas exported. In addition, 113 kg coal will be supplied per day in winter which will last for 4 months; so, the quantity of the coal is 13.6 t/yr.

#### 2.1.2. BPC System

The other biogas project, where degraded material is BPC, is located in the Dongyaozhuang village of Qing country in Hebei province, where crop planting industry is well developed. Because of this, there are a number of types of waste crops straw stalks, and the environment has been polluted by stacking and willfully burning straws. In 1999, the first 1000 m³ CSTR biogas underground tank was built. However, during that period, the technology was immature; so, the biogas tank could not be put into operation. After that, a brick-concrete structure biogas tank of 400 m³ was constructed successfully in 2005, which was the first in China. However, due to limitations of building materials and technology, that biogas project is out of service now. Through summarizing construction operation experiences and improving technology, the new biogas tank of 400 m³ was constructed in 2006. By debugging and running, it was in formal operation in 2007 and has become the star exemplary biogas project in China.

The technology of BPC is up-flow solids reactor (USR), and the material of the tank is welded steel plate. The whole volume of BPC is 400 m³, with treatment capacity of 900 kg corn straw every day. To maintain the feeding concentration, 2~3 t water should be added per day. The fermentation temperature is 44~55°C, and the biogas production capacity is 480 m³/d. The whole biogas system needs 200 m³ of biogas heated per day in winter and 120 m³/d in spring and autumn. The biogas net output amount is 280 m³/d in winter and 360 m³/d in spring and autumn. Because it is not necessary to heat it, the biogas net output amount is 470 m³/d in summer. The same as BPF, 150 kg/d coal is used to heat the biogas tank which lasts about 4 months, and the amount of coal is 18 t/yr.

### 2.2. Emergy Accounting

The input and output raw data of the two biogas systems are recorded for the whole year of 2011. The details of emergy accounting can be referred to through the works of Odum [16]. The process of emergy accounting consists of two steps. The first is to define the boundary of the system and then make emergy evaluation tabulation. The next step is to sum all the emergy contributions from the independent inputs and biogas systems evaluation as shown in the established emergy indicator framework. 

**Figure 2:**
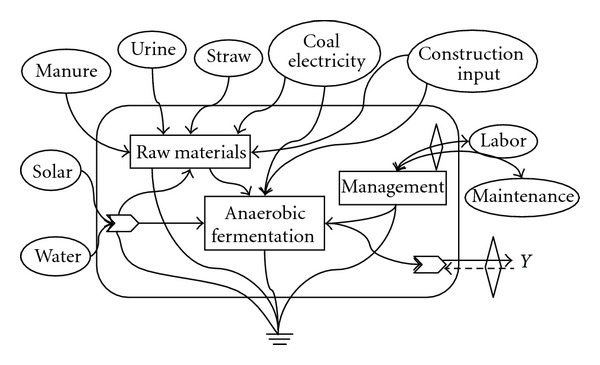
Emergy system diagram of the biogas project.

The emergy system is shown in Figure 2. Input resource is divided into three parts: free local renewable resources (RRs) embracing raw materials (feces/corn straw), solar energy, and underground watering, renewable purchased (RP) inputs includes human labor, and nonrenewable purchased (NP) inputs covering construction inputs, coal and electricity. All the products including biogas and biogas residues are system yield (Y). In the counting process, the whole economic values are converted into solar emergy (sej). And then solar emergy turns into emergy-monetary value (Em$) through an emergy-monetary ratio. In this paper, emergy-monetary value (Em$) represents not only macroeconomy value of emergy flow, but also ecological-economic value, which assesses the eco-efficiency, environmental impact, and the sustainable capacity of the system.

### 2.3. Emergy Evaluation Index

To analyze these two types of biogas systems for the aspects of ecological-economic benefit, environmental benefit, and sustainable development ability, ten evaluation indices are applied in this paper.

#### 2.3.1. Purchase Emergy Ratio (PER)

It is a ratio of input emergy social economy feedback and total input emergy. It depends on system's dependent degree to external resources.

#### 2.3.2. Natural Emergy/Purchase Emergy

This ratio is the emergy from natural resources divided by input emergy social economy feedback, which shows the condition of industrial competitiveness.

#### 2.3.3. Emergy Investment Ratio (EIR)

It's a ratio of input emergy social economy feedback and input emergy of natural resources, which shows the cost of the system. 

#### 2.3.4. Emergy Yield Ratio (EYR)

It is a ratio of the total output emergy and input emergy social economy feedback, which represents the situation of the system production.

#### 2.3.5. Emergy Self-Sufficiency Ratio (ESR)

ESR shows the self-maintenance ability of the system. It is the emergy from nature divided by the total output emergy of the system.

#### 2.3.6. Ratio of Waste Treatment (%W)

It is a ratio of the emergy of waste (for a biogas project, it means the raw materials, such as corn straw and feces) treatment and the total output emergy of the system. This value shows the ability of the treatment of waste of the whole system.

#### 2.3.7. Environmental Loading Ratio (ELR)

This is an important index of environment, [which means the pressure to environment from the system.] It is equal to the ratio of total nonrenewable emergy divided by renewable emergy.

#### 2.3.8. Feedback Yield Ratio (FYR)

This ratio means the emergy of system self-feedback (for biogas system, it is the part of biogas that is heated) divided by economy feedback emergy, which shows the self-organizing ability of the system.

#### 2.3.9. Renewable Ratio (%R)

It is a ratio of renewable emergy and input emergy of the system, which means the system's renewable property.

#### 2.3.10. Emergy Sustainability Ratio (ESR)

This evaluation index shows the situation of the system sustainability, which is equal to EYR divided by ELR.

## 3. Results and Discussion

### 3.1. Emergy Accounting

**Figure 3:**
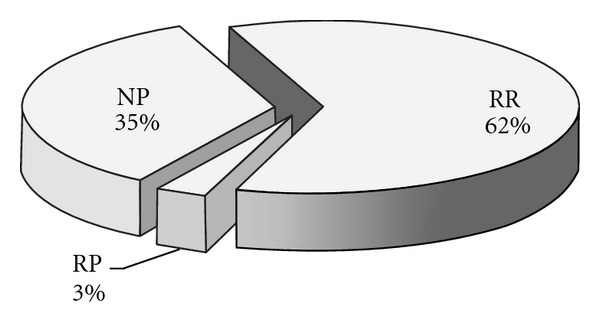
Emergy input ratio of BPC.

**Figure 4:**
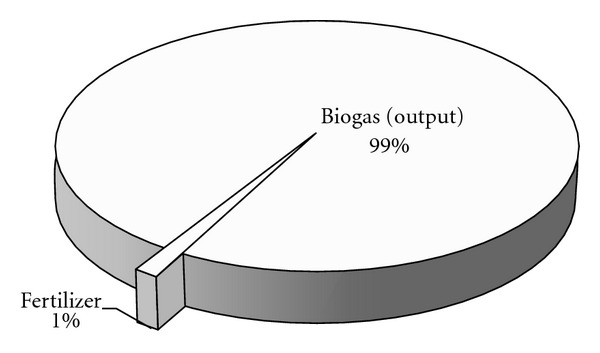
Emergy output ratio of BPC.

As shown in Table 1, the total emergy input of the BPC system amounts to 2.07 × 10^17^ sej/yr, of which the RR, RP and NP contribute 62%, 3%, and 35%, respectively (shown in Figure 3). The emergy yield of the biogas system consists of biogas and biogas residues, and the biogas residues consist of three parts: nitrogen fertilizer, phosphate fertilizer, and potash fertilizer. As shown in Figure 4, biogas is the most important production, the yield of which amounts to 6.92 × 10^17^ sej/yr and makes up 99% of the total emergy yield. The part of the biogas residues is only 1%.

**Figure 5:**
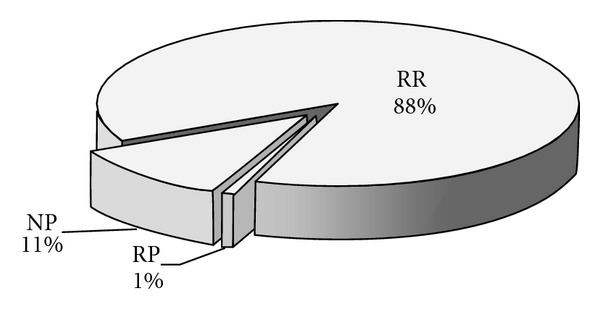
Emergy input ratio of BPF.

**Figure 6:**
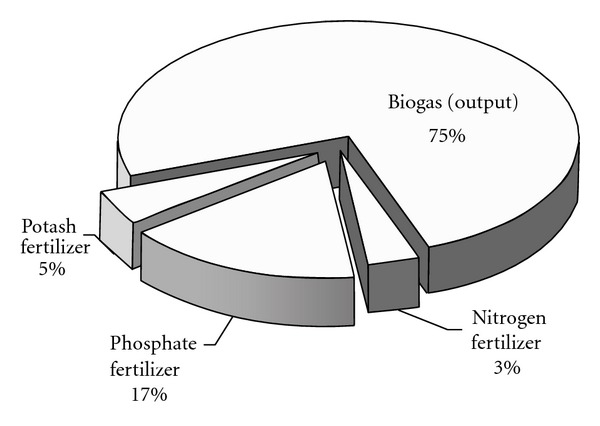
Emergy output ratio of BPF.

Based on the data in Table 2, the total emergy input of BPF system amounts to 6.42 × 10^17^ sej/yr, of which RR, RP, and NP contribute 88%, 1%, and 11%, respectively (Figure 5). As to RR, solid manure and urine (including flushing sewage) account for 46.50% and 53.50%, respectively. The same as BPC system, the emergy yield of the biogas system also consists of biogas and biogas residues. As shown in Figure 6, biogas is the most important production, the yield of which amounts to 8.16 × 10^17^ sej/yr and makes up 75% of the total emergy yield. The remaining part of the biogas residues is divided into three parts: nitrogen fertilizer (3%), phosphate fertilizer (17%), and potash fertilizer (5%).

In comparison to BPC system, the input emergy of BPF system is 4.38 × 10^17^ sej/yr more than BPC, because RR is a large proportion of BPF system. Otherwise, as to output emergy of BPC system, biogas output is 99%, and biogas manure is 1%; concerning output emergy of BPF system, biogas output is 75%, and biogas manure is 25%. The reason is the difference of the nature of digestion materials, construction scale, and digestion technology. For the BPC system, the greatest goal is biogas production; so, we call this kind of biogas project energy-ecology type. On the other hand, BPF system realizes the target of dealing with manure and wastewater efficiently, which is inclined to the energy-environmental type.

### 3.2. Ecological-Economic Analysis

A biogas project is one of the circular agriculture ecology patterns dealing with agricultural wastes, and the ecological-economic benefits could be measured by emergy-monetary value, which is a specific form of emergy-monetary value reflected in the economic market.

In Table 3, the total investment funds of BPF and BPC are $15,700/yr and $21,500/yr, respectively. The BPF resources (feces) do not need to be bought; so, investment funds of the BPF system include RP and NP, but those of BPC system include RR, RP, and NP. Thus, the BPF system can alleviate the government pressure and be worthy of widely promotion. In contrast, the BPC resources (corn straw) need to be bought, because of its extensive application, such as fuel to heat water and briquetting forming fuel. In addition, straw must be crushed before anaerobic digestion. And supplementary additives, such as protein powder, are required in the anaerobic digestion process. So, the RP and NP of the BPC system are both higher than those of the BPF. Moreover, the BPF system has solar heating system which decreases the quantity of coal and electricity; so, the whole input energy is less than BPC.

The outputs of BPF and BPC system are $44,000/yr and $29,600/yr, respectively. The main reason is the large gap of output economic value of biogas residues, which causes differences between the two biogas projects technologies. The ecological-economic values of BPF and BPC system are $28,300/yr and $8,100/yr, respectively. Because both of the materials are renewable natural resources, the two biogas projects have high ecological-economic values and significant ecological environmental benefits. Most biogas from the project can meet the energy needs; biogas manure can be used as organic manure for crops, vegetables, and fruits. So they all improve the ecological-economy value.

### 3.3. Emergy Evaluation Index Analysis

All the emergy evaluation indices are classified by ecological-economic benefit, environmental benefit, and sustainable development ability (shown in Table 4).

#### 3.3.1. Ecological-Economic Benefit

PER, natural emergy/purchase emergy, EIR, and ESR of BPF system are 0.32, 4.49, 0.22, and 1.42 times more than BPC system, respectively. This indicates that the BPF system depends less on renewable resources and has greater competitiveness and higher natural resources utilization efficiency and self-sufficiency ability than BPC systems. This is because BPC's material, corn straw, needs to be purchased, but BPF's material, feces, does not need fund support.

As to the net contribution of economic, although the feedback emergy of the two systems appears to be the same, the EYR of BPF system is about 1.57 times more than that of BPC systems, because the BPF system has much more yield emergy, which means that the BPF system has low cost, good production efficiency, high emergy utilization efficiency, and competitiveness. 

Considering all the previous indices, BPF system has higher ecological-economic benefit than BPC system.

#### 3.3.2. Environmental Benefit

The %W of BPF system is about 1.43 times more than that of BPC systems. Raw materials of BPF system are livestock manure, poultry and urine. And the total fermentation volume is 624 m^3^, which consists of a primary fermentation reactor (400 m^3^) and a secondary fermentation reactor (224 m^3^). On the other hand, the raw material of BPC system is only straw, with fermentation volume of 480 m^3^. The wastes treatment quantities of the BPF system and BPC system converted into solar energy value are 5.67 × 10^17^ sej/yr and 1.27 × 10^17^ sej/yr, which means that BPF system, has higher degradation efficiency. 

ELR is an important index which reflects the degree of influence of the system on the environment. The ELRs of BPF and BPC system are 0.13 and 0.53, which are both less than 1. The results show that the two systems have a small impact on environment. However, owing to the higher renewable emergy ratio and more wastes treatment quantity, BPF system has much higher environmental benefit.

#### 3.3.3. The Analysis of Sustainable Development Ability

To analyze sustainable development ability, there are three parts to be considered: FYR, %R, and ESI.

The first index is FYR. FYR is the ratio of the emergi of system self-feedback (for a biogas system, it is the part of biogas heated) divided by economy feedback emergy. The economic feedback emergies of BPF and BPC are 7.81 × 10^16^ sej/yr and 7.92 × 10^16^ sej/yr, while the self-feedback emergies are 3.97 × 10^17^ sej/yr and 2.05 × 10^17^ sej/yr. The previous results are the result of the quantity of energy used to heat 400 m³ primary fermentation reactor and 224 m³ secondary fermentation reactors. The FYR of BPF is 1.96 times more than BPC, which means that the ability of BPF's self-organizing is much better than BPC's.

Another index is %R, which indicates the renewable character of the biogas system. The renewable energy and the emergy devoted to BPF are both more than the BPC system. At the same time, %R of BPF is 1.36 times more than the BPC system. The results show that the ability of the system renewable character of BPF is better.

The last index, ESI, states the relation between the environment and the emergy produced. A perfected biogas system has not only high output, but also a far-reaching influence. The ESI of BPF is 6.61 times more than the BPC system, which shows that the BPF system has bright sustainable development possibilities.

## 4. Conclusion

The large- and medium-sized biogas projects where materials are corn straw and feces both have high ecological-economic benefit. In this research, the ecological-economic values of BPF system and BPC system are $28,300/yr and $8,100/yr, respectively. According to the monetary, environment, and human production factors, both of the two types of biogas projects own great development potential.

As to the whole input emergy, renewable natural resources take the largest percentage, which means that both of the two systems have good treatment ability of agricultural waste (feces and crop straw). In the BPC system, the amount of biogas output is much more than biogas residues. With the rapid development of biogas projects, recycling treatment of waste and sewage has become a hot topic, and the comprehensive utilization of biogas residues has become a primary goal. Therefore, technology needs to be improved in order to utilize biogas residues efficiently.

From the analysis of the point of emergy, BPF system is superior to BPC system in the aspects of ecological economic benefit, environmental benefit and the sustainable development ability. This result is due to problems of the difficulty of corn straw seasonal collection and degradation. As to this situation, BPC system can be considered if anaerobic digestion is combined with other raw materials, such as poultry and livestock manure, urine and household garbage.

## Figures and Tables

**Table 1:** Energy accounting for BPC system.

No.	Item^a^	Units	Raw date	Transformity	References	Solar emergy	Em
				(sej/unit)		(sej/yr)	($)
*Local renewable resources *							
1	Underground water	J	3.65*E* + 09	4.10*E* + 04	Odum, 1996 [16]	1.50*E* + 14	0
2	Corn straw	J	4.72*E* + 12	2.70*E* + 04	Odum, 1996 [16]	1.27*E* + 17	3180

Total (RR)						1.28*E* + 17	3180

*Renewable purchased inputs *							
3	Human labor	J	1.84*E* + 10	3.80*E* + 05	Xi and Qin, 2006 [17]	6.99*E* + 15	5804

Total (RP)						6.99*E* + 15	5804

*Nonrenewable purchased inputs *							
4	Steel plate^b^	US $	1.75*E* + 06	1.40*E* + 09	Brown and Ulgiati, 2004 [18]	2.45*E* + 15	846
5	Equipments, PE pipes^b^	US $	5.44*E* + 03	4.94*E* + 12	Brown and Ulgiati, 2004 [18]	2.69*E* + 16	5441
6	Civil work cost^b^	US $	6.05*E* + 02	4.94*E* + 12	Xi and Qin, 2006 [17]	2.99*E* + 15	605
7	Maintenance cost	US $	6.05*E* + 02	4.94*E* + 12	Xi and Qin, 2006 [17]	2.99*E* + 15	605
8	Coal	J	4.82*E* + 11	4.00*E* + 04	Odum, 1996 [16]	1.93*E* + 16	2177
9	Electricity	J	1.08*E* + 11	1.59*E* + 05	Xi and Qin, 2006 [17]	1.72*E* + 16	1886
10	Protein powder	US $	9.27*E* + 01	4.94*E* + 12	Xi and Qin, 2006 [17]	4.58*E* + 14	931

Total (NP)						7.22*E* + 16	12491

Total input						2.07*E* + 17	21475

*System feedback *							
11	Biogas (heated)	J	8.28*E* + 11	2.48*E* + 05	Bastianoni and Marchettini, 2000 [15]	2.05*E* + 17	7666

Total (F)						2.05*E* + 17	7666

*Yield *							
12	Biogas (output)	J	2.79*E* + 12	2.48*E* + 05	Bastianoni and Marchettini, 2000 [15]	6.92*E* + 17	28996
13	Nitrogen fertilizer	g	6.58*E* + 05	4.62*E* + 09	Zhang et al., 2005 [19]	3.04*E* + 15	145
14	Phosphate fertilizer	g	3.29*E* + 05	1.78*E* + 10	Zhang et al., 2005 [19]	5.86*E* + 15	218
15	Potash fertilizer	g	4.94*E* + 05	2.96*E* + 09	Zhang et al., 2005 [19]	1.46*E* + 15	193

Total yield (Y)						7.02*E* + 17	29553

^a^Local nonrenewable resources can be neglected.

^b^Items have been divided by a lifetime of 20 years.

**Table 2:** Energy accounting for BPF system.

No.	Item^a^	Units	Raw date	Transformity	References	Solar emergy	Em
				(sej/unit)		(sej/yr)	($)
*Local renewable resources *							
1	Sunlight	J	1.26*E* + 11	1.00*E* + 00	Odum, 1996 [16]	1.26*E* + 11	0
2	Solid manure	J	9.76*E* + 12	2.70*E* + 04	Odum, 1996 [16]	2.64*E* + 17	0
3	Feces	J	7.98*E* + 10	3.80*E* + 06	Odum, 1996 [16]	3.03*E* + 17	0

Total (RR)						5.67*E* + 17	0

*Renewable purchased inputs *							
4	Human labor	J	1.38*E* + 10	3.80*E* + 05	Xi and Qin, 2006 [17]	5.24*E* + 15	4353

	Total (RP)					5.24*E* + 15	4353

*Nonrenewable purchased inputs *							
5	Civil work cost^b^	US $	2.96*E* + 03	4.94*E* + 12	Xi and Qin, 2006 [17]	1.46*E* + 16	2963
6	Equipments, PE pipes^b^	US $	5.20*E* + 03	4.94*E* + 12	Xi and Qin, 2006 [17]	2.57*E* + 16	5200
7	Appurtenant work^b^	US $	7.10*E* + 02	4.94*E* + 12	Xi and Qin, 2006 [17]	3.51*E* + 15	713
8	Maintenance cost	US $	6.05*E* + 02	4.94*E* + 12	Xi and Qin, 2006 [17]	2.99*E* + 15	605
9	Coal	J	3.64*E* + 11	4.00*E* + 04	Odum, 1996 [16]	1.46*E* + 16	738
10	Electricity	J	7.28*E* + 10	1.59*E* + 05	Xi and Qin, 2006 [17]	1.16*E* + 16	1173

Total (NP)						7.29*E* + 16	11391

Total input						6.45*E* + 17	15744

*System feedback *							
11	Biogas (heated)	J	1.60*E* + 12	2.48*E* + 05	Bastianoni and Marchettini, 2000 [15]	3.97*E* + 17	13881

Total (F)						3.97*E* + 17	13881

*Yield *							
12	Biogas (output)	J	3.29*E* + 12	2.48*E* + 05	Bastianoni and Marchettini, 2000 [15]	8.16*E* + 17	28561
13	Nitrogen fertilizer	g	8.63*E* + 06	4.62*E* + 09	Zhang et al., 2005 [19]	3.99*E* + 16	1838
14	Phosphate fertilizer	g	1.02*E* + 07	1.78*E* + 10	Zhang et al., 2005 [19]	1.82*E* + 17	6844
15	Potash fertilizer	g	1.73*E* + 07	2.96*E* + 09	Zhang et al., 2005 [19]	5.12*E* + 16	6796

Total yield (Y)						1.09*E* + 18	44039

^a^Local nonrenewable resources can be neglected.

^b^Items have been divided by a lifetime of 20 years.

**Table 3:** Table of emergy monetary value $/yr.

Item	BPC	BPF
RR	3.18 × 10^3^	/
RP	5.80 × 10^3^	4.35 × 10^3^
NP	1.25 × 10^4^	1.14 × 10^4^
Total input	2.15 × 10^4^	1.57 × 10^4^
Biogas (output)	2.90 × 10^4^	2.86 × 10^4^
Nitrogen fertilizer	1.45 × 10^2^	1.84 × 10^3^
Phosphate fertilizer	2.18 × 10^2^	6.84 × 10^3^
Potash fertilizer	1.93 × 10^2^	6.80 × 10^3^
Total yield	2.96 × 10^4^	4.40 × 10^4^
Ecological-economic value	0.81 × 10^4^	2.83 × 10^4^

**Table 4:** The comparative table of energy index.

Item	Evaluation index	BPC	BPF
Ecological-economy benefit	Purchased emergy ratio (PER)	0.38	0.12
	Natural emergy/purchased emergy	1.62	7.26
	Emergy investment rate (EIR)	0.62	0.14
	Emergy yield ratio (EYR)	8.86	13.95
	Emergy self-sufficiency ratio (ESR)	0.62	0.88

Environmental benefit	Waste processing ratio (%W)	61.30	87.89
	Environmental load ratio (ELR)	0.53	0.13

Sustainable development	Feedback yield ratio (FYR)	2.59	5.08
	Renewable ratio (%R)	65.15	88.70
	Sustainability index (ESI)	16.57	109.50

## Appendices

### A.

Notes to Table 1:

1. Underground water
  Number: 2500 kg/d
  Standard energy value: 4 kJ/ kg
  Energy: 2500 kg/d × 4 × 10^3^ J/kg × 365 d/yr = 3.65 × 10^9^ J/yr
2. Corn straw
  Number: 900 kg/d
  Standard energy value: 14355.72 kJ/kg
  Energy: 900 kg/d × 14355.72 × 10^3^ J/kg × 365 d/yr = 4.72 × 10^12^ J/yr
3. Human labor
  Days: 4 × 365 d/yr = 1460 d/yr
  Standard energy value: 12600 kJ/d
  Energy: 1460 d/yr × 12600 kJ/d = 1.84 × 10^10^ J/yr
4. Steel plate
  Number: 3.5 × 10^7^ g/20 yr = 1.75 × 10^6^ g/yr
5. Equipments, PE pipes
  Number: (900000/20 yr)RMB/8.27 = 5.44 × 10^3^ (2000 US$)
6. Civil work cost
  Number: (100000/20 yr)RMB/8.27 = 6.05 × 10^2^ (2000 US$)
7. Maintenance cost
  Number: 5000 RMB/8.27 = 6.05 × 10^2^ (2000 US$)
8. Coal
  Number: 18000 kg/yr
  Standard energy value: 26777.6 kJ/kg
  Energy: 18000 kg/yr × 26777.6 kJ/kg = 4.82 × 10^11^ J/yr
9. Electricity
  Number: 30000 kw*·*h/yr
  Standard energy value: 3598.24 kJ/kw*·*h
  Energy: 30000 kw*·*h/yr × 3598.24 kJ/kw*·*h = 1.08 × 10^11^ J/yr
10. Protein powder
  Number: (182.5 kg/yr × 4.2 RMB/kg)/8.27= 9.27 × 10^1^ (2000 US$)
11. Biogas (heated)
  Number: 39600 m^3^/yr
  Standard energy value: 20920 KJ/m^3^
  Energy: 39600 m^3^/yr × 20920 KJ/m^3^ = 8.28 × 10^11^ J/yr
  Number: 76500 m^3^/yr
  Standard energy value: 20920 kJ/m^3^
  Energy: 76500 m^3^/yr × 20920 kJ/m^3^ = 1.60 × 10^12^ J/yr
12. Biogas (output)
  Number: 133200 m^3^/yr
  Standard energy value: 20920 kJ/m^3^
  Energy: 133200 m^3^/yr × 20920 kJ/m^3^ = 2.79 × 10^12^ J/yr
  Number: 157500 m^3^/yr
  Standard energy value: 20920 kJ/m^3^
  Energy: 157500 m^3^/yr × 20920 kJ/m^3^ = 3.29 × 10^12^ J/yr
13. Nitrogen fertilizer
  Number: 6.58 × 10^5^ g/yr
14. Phosphate fertilizer
  Number: 3.29 × 10^5^ g/yr
15. Potash fertilizer
  Number: 4.94 × 10^5^ g/yr

### B.

Notes to Table 2:

1. Solar energy
  Standard energy value: 4.1868 × 10^3^ J/kg*·*k
  Energy: 50 m^3^/d × 10^3^ Kg/m^3^ × 120 d/yr × 4.1868 × 10^3^ J/(Kg*·*k) × 5 k = 1.26 × 10^11^ J/yr
2. Solid manure
  Number: 4.015 × 10^6^ kg/yr
  Total solid: 18%
  Standard energy value: 13500 kJ/kg
  Energy: 4.015 × 10^6^ kg/yr × 18% × 13500 kJ/kg = 9.76 × 10^12^ J/yr
3. Urine (including flushing sewage)
  Number: 12410 m^3^/yr
  Standard energy value: 6.43 × 10^6^ J/m^3^
  Energy: 12410 m^3^/yr × 6.43 × 10^6^ J/m^3^ = 7.98 × 10^10^ J/yr
4. Human labor
  Days: 3 × 365 d/yr = 1095 d/yr
  Standard energy value: 12600 kJ/d
  Energy: 1095 d/yr × 12600 kJ/kg = 1.38 × 10^10^ J/yr
5. Civil work cost
  Number: (734000/30 yr)RMB/8.27= 2.96 × 10^3^ (2000 US$)
6. Equipments, PE pipes
  Number: (1289000/30 yr)RMB/8.27 = 5.20 × 10^3^ (2000 US$)
7. Appurtenant work
  Number: (176000/30 yr)RMB/8.27 = 7.10 × 10^2^ (2000 US$)
8. Maintenance cost
  Number: 5000 RMB/8.27 = 6.05 × 10^2^ (2000 US$)
9. Coal
  Number: 13600 kg/yr
  Standard energy value: 26777.6 k J/kg
  Energy: 13600 kg/yr × 26777.6 kJ/Kg = 3.64 × 10^11^ J/yr
10. Electricity
  Number: 20221 kw*·*h
  Standard energy value: 3598.24 kJ/kw*·*h/yr
  Energy: 20221 KW*·*h/yr × 3598.24 kJ/kw*·*h = 7.28 × 10^10^ J/yr
11. Biogas (heated)
  Number: 76500 m^3^/yr
  Standard energy value: 20920 kJ/m^3^
  Energy: 76500 m^3^/yr × 20920 kJ/m^3^ = 1.60 × 10^12^ J/yr
12. Biogas (output)
  Number: 157500 m^3^/yr
  Standard energy value: 20920 kJ/m^3^
  Energy: 157500 m^3^/yr × 20920 kJ/m^3^ = 3.29 × 10^12^ J/yr
13. Nitrogen fertilizer
  Number: 8.63 × 10^3^ kg/yr
14. Phosphate fertilizer
  Number: 1.02 × 10^4^ kg/yr
15. Potash fertilizer
  Number: 1.73 × 10^4^ kg/yr.
